# We Do Not Know Whether Head‐Up Sleep Enhances Erythropoiesis

**DOI:** 10.1111/apha.70282

**Published:** 2026-07-26

**Authors:** Meihan Guo, David Montero

**Affiliations:** ^1^ Department of Medicine Beth Israel Deaconess Medical Center, Harvard Medical School Boston Massachusetts USA; ^2^ Faculty of Medicine School of Public Health, Hong Kong University Hong Kong Hong Kong; ^3^ Department of Medicine School of Clinical Medicine, Hong Kong University Hong Kong Hong Kong; ^4^ Libin Cardiovascular Institute of Alberta, University of Calgary Calgary Alberta Canada

Red blood cells fill the “holy grail” of endurance performance. The more red blood cells in the circulation, the greater the capacity to deliver O_2_ to active muscle fibers, which determines peak O_2_ consumption (VO_2peak_). Elite endurance athletes, without exception, present with a higher number of red blood cells per unit of body weight than untrained individuals. Beyond genetics, what increases the production of red blood cells, that is, erythropoiesis? Two main stimuli are established to date, chronic hypoxia and endurance training. We hypothesized an additional stimulus: chronic low circulating blood volume (BV) in the thoracic cavity, leading to neurohumoral reflexes increasing the production of erythropoietin (EPO) [1].

We tested the hypothesis that head‐up sleep (HUS, 40 cm elevation of bed head, 12° head‐up tilt) for 12 weeks would increase intravascular volumes, including plasma volume, total red blood cell volume (RBCV), and hemoglobin mass (Hb_mass_) in healthy nonobese and nonsmoking women and men (42 ± 18 years, 51% women) [2]. The participants were randomly allocated to (1) increased fluid (water) intake (IFI, 42 mL of water·kg^−1^) (*n* = 24), or (2) HUS + IFI (*n* = 11). The IFI condition was intended to control for the placebo effect: the potential belief that the intervention elicited beneficial effects. Both groups were matched by sex, age, body composition, physical activity, and VO_2peak_. Three individuals withdrew from the IFI intervention due to a busy working schedule or dislike of increased urination. Two individuals withdrew from the HUS + IFI intervention due to travel or sleep disturbance. Water intake was similar in IFI and HUS + IFI interventions (46.8 ± 4.8 vs. 44.5 ± 6.1 mL·kg^−1^, *p* = 0.248). Heart rate, monitored at night, was elevated during the initial hours of sleep in the HUS + IFI group. Intravascular volumes and Hb_mass_ were unchanged with IFI or HUS + IFI (*p* ≥ 0.246). At rest, cardiac filling and output per heart beat (stroke volume) were increased with both interventions (*p* ≤ 0.007); the effect on resting stroke volume was larger with HUS + IFI than IFI alone (*p* = 0.037). During exercise, peak cardiac output and VO_2peak_ were not altered with IFI or HUS + IFI, due to reduced left ventricular emptying (*p* ≤ 0.003). The main results of this study are outlined in Figure 1. Our hypothesis was disproved. Could the control condition for the placebo effect (increased water intake) prevent the enhancement of erythropoiesis with HUS? We do not know, but to date, there is no evidence of increased water intake leading to reduced Hb_mass_, rather the opposite [3].

**FIGURE 1:**
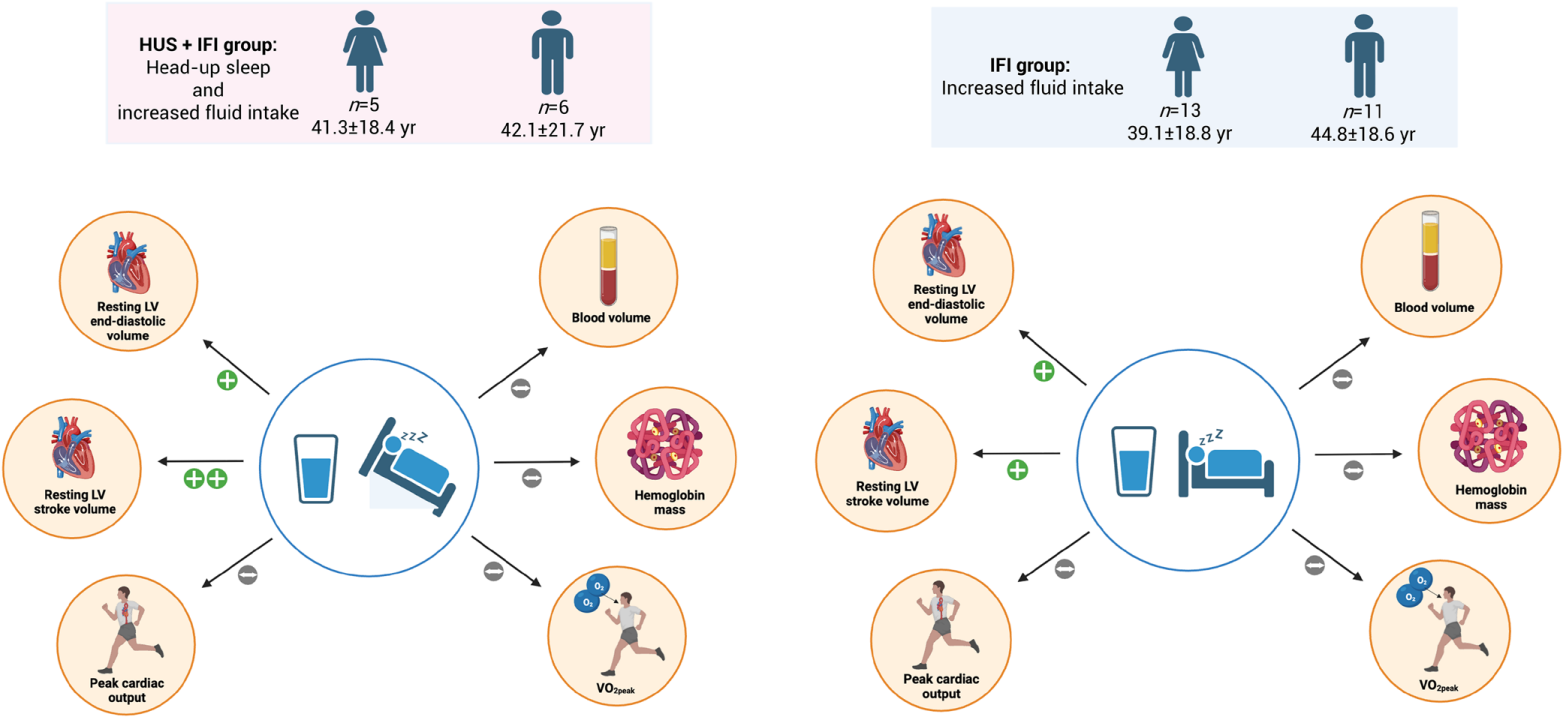
Effects of head‐up sleep with increased fluid (water) intake (HUS + IFI) or IFI alone on blood volume, hemoglobin mass, resting cardiac volumes, cardiac and aerobic capacities. HUS + IFI, head‐up sleep and increased fluid intake; IFI, increased fluid intake; LV, left ventricle; VO_2peak_, peak oxygen consumption.

A recent study, not manipulating water intake, assessed the effects of 5 weeks of HUS (6° head‐up tilt) on Hb_mass_ in healthy young men (*n* = 9, 24 ± 5 years) [4]. No hemodynamic measure was taken during the home‐based HUS intervention, but time in bed and sleep quality were not altered by HUS. Arterial blood pressure was unchanged by HUS, as measured before rising from bed after sleeping in the laboratory for two nights. The positive result was a small increase in Hb_mass_ (36 g, +4%) with HUS. The authors concluded: HUS promotes erythropoiesis and may be an adjunct treatment for patients with anemia [4].

Besides the sparse evidence available, the following points in the previous study must be considered before reaching conclusions:

1. The placebo effect was not controlled [4]. It is plausible that young men willing to participate in a study entailing a change in their sleep habits for 5 weeks were informed of the potential benefits of HUS.
2. Nearly all individuals (eight of nine) did increase Hb_mass_ with HUS [4]. The authors typically report a lower percentage of responders to interventions of similar duration (5 weeks) and a similarly small average gain in Hb_mass_ (+30 to 42 g of Hb_mass_) [5, 6, 7, 8]. No information is available on the number of initially recruited subjects and those excluded and/or lost to follow‐up (if any).
3. As for their rationale, the authors state that a reduction in central venous pressure induced by 3 h of head‐up tilt increases circulating EPO [4]. They cite a study in which we observed a slight increase in EPO with head‐up tilt if no control group is included in the analyses [1]. When compared with the opposite condition, that is, head‐down tilt, EPO was not increased (*p* = 0.160) [1]. Likewise, EPO is not increased by HUS in their study [4].
4. The increment in circulating Hb_mass_ (+4%) should have increased aerobic exercise capacity and performance [4, 9]. No measure of exercise capacity or performance is reported [4].
5. The same increment in Hb_mass_ (+4%) has been observed with 4 days of the opposite stimuli, head‐down bed rest, indicating a potential non‐specificity of a small hematological response [10].
6. There is no evidence of any degree of secondary polycythemia in multiple populations in which HUS is prescribed.

Considering the available evidence, we conclude the obvious: more research is needed.

## Author Contributions

D.M. conceived the article. D.M. and M.G. drafted the manuscript. M.G. and D.M. critically revised the manuscript and provided the final approval.

## Funding

This work was funded by the Research Grants Council of Hong Kong (106210224, to D.M.).

## Conflicts of Interest

The authors declare no conflicts of interest.

## Data Availability

The data that support the findings of this study are available from the corresponding author upon reasonable request.
